# Structure aided design of a Neu5Gc specific lectin

**DOI:** 10.1038/s41598-017-01522-9

**Published:** 2017-05-04

**Authors:** Christopher J. Day, Adrienne W. Paton, Melanie A. Higgins, Lucy K. Shewell, Freda E.-C. Jen, Benjamin L. Schulz, Brock P. Herdman, James C. Paton, Michael P. Jennings

**Affiliations:** 1 Institute for Glycomics, Griffith University, Gold Coast, QLD 4222 Australia; 2 Research Centre for Infectious Diseases, Department of Molecular and Cellular Biology, University of Adelaide, S.A., 5005 Australia; 3 Australian Infectious Diseases Research Centre, School of Chemistry and Molecular Biosciences, The University of Queensland, St. Lucia Brisbane, QLD 4072 Australia

## Abstract

Subtilase cytotoxin (SubAB) of *Escherichia coli* is an AB5 class bacterial toxin. The pentameric B subunit (SubB) binds the cellular carbohydrate receptor, α2–3-linked *N*-glycolylneuraminic acid (Neu5Gc). Neu5Gc is not expressed on normal human cells, but is expressed by cancer cells. Elevated Neu5Gc has been observed in breast, ovarian, prostate, colon and lung cancer. The presence of Neu5Gc is prognostically important, and correlates with invasiveness, metastasis and tumour grade. Neu5Gc binding by SubB suggests that it may have utility as a diagnostic tool for the detection Neu5Gc tumor antigens. Native SubB has 20-fold less binding to N-acetlylneuraminic acid (Neu5Ac); over 30-fold less if the Neu5Gc linkage was changed from α2–3 to α2–6. Using molecular modeling approaches, site directed mutations were made to reduce the α2–3 ${\boldsymbol{\gg }}$ α2–6-linkage preference, while maintaining or enhancing the selectivity of SubB for Neu5Gc over Neu5Ac. Surface plasmon resonance and glycan array analysis showed that the SubBΔS106/ΔT107 mutant displayed improved specificity towards Neu5Gc and bound to α2–6-linked Neu5Gc. SubBΔS106/ΔT107 could discriminate NeuGc- over Neu5Ac-glycoconjugates in ELISA. These data suggest that improved SubB mutants offer a new tool for the testing of biological samples, particularly serum and other fluids from individuals with cancer or suspected of having cancer.

## Introduction

AB5 toxins exert their effects in a two-step process: (i) binding of the pentameric B subunit to specific glycan receptors on the target cell surface; (ii) internalisation of the AB5 toxin, followed by A subunit-mediated inhibition or corruption of essential host functions^1^. The B subunits of AB5 toxins recognize cell surface glycan receptors, directing internalization and intracellular trafficking of the holotoxin. Specificity of these protein-glycan interactions is critical for pathogenesis, as it determines host susceptibility and tissue tropism. Moreover, the pentavalent interactions between AB5 toxin B subunits and their cognate glycans result in very high affinity binding, making them powerful ligands for glycan detection, a noteworthy example being use of the cholera toxin B subunit for detection of the ganglioside GM1 in histopathological sections^2^ and for labelling of lipid rafts in membranes^3^.

In 2004 Paton *et al*. described the discovery and initial biological characterization of a new sub-family of bacterial AB5 toxins with the prototype termed subtilase cytotoxin (SubAB)^4^. In the case of SubAB, the A subunit (SubA) was found to be a subtilase family serine protease with exquisite specificity for the essential endoplasmic reticulum chaperone BiP/GRP78^5^. Structural studies revealed that unlike most subtilases, SubA possessed an unusually deep active site cleft, explaining its exquisite substrate specificity^5^. SubA has proven to be a powerful tool for examining the role of BiP in diverse cellular processes and it also has potential as a cancer therapeutic^6, 7^. Significantly, glycan array analysis has shown that the B subunit of the toxin (SubB) has a high degree of binding specificity for glycans terminating with α2–3-linked *N*-glycolylneuraminic acid (Neu5Gc), a sialic acid that humans cannot synthesise^8^. Of all the glycans on the array, the best binding occurred with Neu5Gcα2–3 Galβ1–4GlcNAcβ-. Binding of labelled toxin to the array was reduced 20-fold if the Neu5Gc was changed to Neu5Ac; over 30-fold if the Neu5Gc linkage was changed from α2–3 to α2–6; and 100-fold if the sialic acid was removed. The overall pattern of binding to structures represented on the array indicated that SubB has a high affinity for terminal α2–3-linked Neu5Gc with little discrimination for the penultimate moiety. The crystal structure of the SubB-Neu5Gc complex revealed the basis for this specificity. The additional hydroxyl on the methyl group of the *N*-acetyl moiety that distinguishes Neu5Gc from Neu5Ac interacts with Tyr78^OH^ of SubB and hydrogen bonds with the main chain of Met10^8^. These key interactions could not occur with Neu5Ac, thus explaining the marked preference for Neu5Gc. Guided by the structural data, key residues were mutagenized in the predicted binding pocket, and this abrogated glycan recognition, cell binding and toxicity. SubB amino acids S12 and Y78 form crucial stabilizing bonds with Neu5Gc^8^. An S12A mutation abolished glycan binding completely, while a Y78F mutation that prevents interactions with the C^11^ OH group that distinguishes Neu5Gc from Neu5Ac reduced glycan binding by 90% and abolished preference of the mutant SubB protein for Neu5Gc over Neu5Ac^8^.

Interestingly, the most prominent form of aberrant glycosylation in human cancers is the expression of glycans terminated by Neu5Gc. Neu5Gc is not expressed in significant levels on normal healthy human cells^9–12^ as humans cannot synthesise Neu5Gc due to an inactivating mutation in the CMAH gene^13^. Nevertheless, research suggests that Neu5Gc presentation in cancer patients can be explained by Neu5Gc absorption through dietary intake of red meat and dairy products, which are the richest sources of Neu5Gc^14^. The presence of Neu5Gc is prognostically important, because its expression frequently correlates with invasiveness, metastasis and the tumour grade^10^. Preferential display of Neu5Gc glycans on cancer cells may be at least partly explained by the hypoxic tumour environment, which markedly induces expression of the sialic acid transporter sialin, resulting in increased display of Neu5Gc and other sialic acids on the cell surface^15^. Due to the fact that sialyl-conjugates regulate adhesion and promote cell mobility, such alterations in surface sialylation may influence the colonisation and metastatic potential of tumour cells^16^. Elevated levels of abnormal sialic acids such as Neu5Gc have been observed in breast, ovarian, prostate, colon and lung cancer^11, 12^. Importantly, incorporation of Neu5Gc in cancer cells is most prominent in soluble glycoproteins found both in the extracellular space and inside the cell, and Neu5Gc is the dominant sialic acid in glycoproteins secreted from cancer cells into the surrounding tissues^9^. The expression of Neu5Gc in cancer is also known to drive production of xenoautoantibodies against Neu5Gc^17, 18^. These anti-Neu5Gc antibodies are being investigated to determine their potential for novel diagnostics, prognostics, and therapeutics in human carcinomas^17^.

Due to its known involvement in cancer and its normally low level in non-cancerous human tissues, detection of high levels of Neu5Gc in serum and in tissues would be considered abnormal and would be indicative of the presence of a tumour. This raises the possibility of exploiting the specificity of SubB for Neu5Gc to develop a high-throughput diagnostic screening test for a range of cancers. However, the poor affinity for α2–6 linked Neu5Gc might impact on the sensitivity of such a test. In the present study, we have examined the interaction between SubB and glycans terminating in either α2–3-linked, or α2–6 linked, Neu5Gc, with a view to designing a SubB mutant with capacity to recognise both types of structures with high affinity.

## Results

### Structure-guided mutation of the glycan binding site of SubB

**Figure 1:**
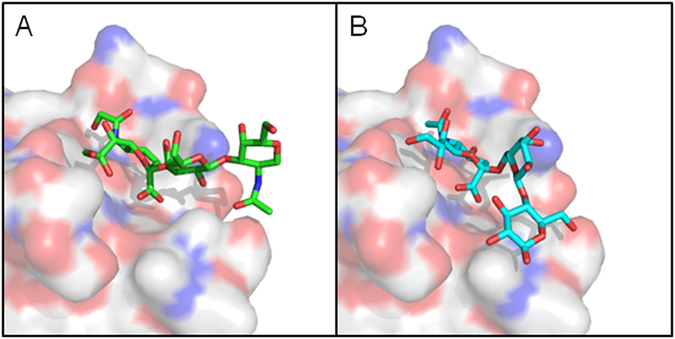
Surface representation of SubB in complex with (**A**) Neu5Gcα2–3Galβ1–3GlcNAc (determined from a X-ray crystal structure (Byres *et al*.^8^)) and (**B**) Neu5Gcα2–6Galβ1–3Glc (modeled with the X-ray crystal structure). Trisaccharides are shown as a green or cyan stick with red and blue residues representing oxygen and nitrogen, respectively.

In order to understand the molecular basis for the preference for α2–3-linked structures, we have compared the interaction between SubB and Neu5Gcα2–3Galβ1–3GlcNAc **(**determined by X-ray crystallography) vs Neu5Gcα2–6 Galβ1–3Glc (Fig. 1). Whereas the sub-terminal sugars of the former glycan extend freely out into the solvent, as reported previously^8^, the tertiary sugar of the α2–6 structure is folded back onto the SubB surface, making close contact with a loop comprising SubB residues T104-E108. This loop is stabilised by a disulphide bond between C103 and C109. The resultant steric hindrance distorts the docking of the terminal Neu5Gc into the binding pocket, accounting for the significantly poorer binding of α2–6-linked Neu5Gc structures observed on the original glycan array analysis.

**Figure 2:**
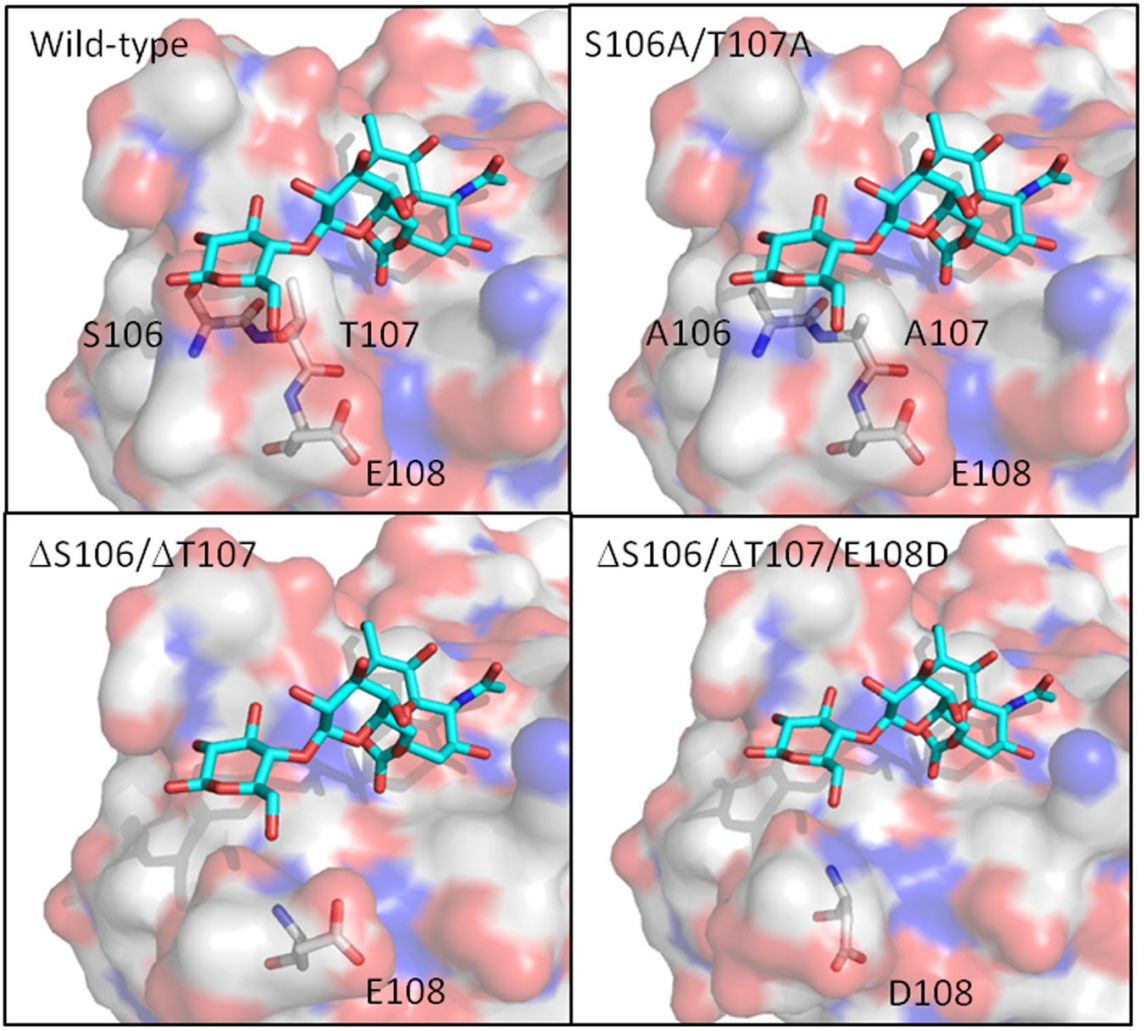
Surface representation of the wild-type and SubB mutants modeled with Neu5GCα2–6Galβ1–3Glc (shown as a cyan stick). The mutated SubB residues are shown as grey sticks and red and blue residues represent oxygen and nitrogen, respectively.

Since α2–6-linked sialic acids are common markers of colon cancer^19, 20^ and are linked to prognosis in a range of cancers^21^, we used molecular engineering to improve binding of α2–6-linked Neu5Gc structures to SubB by designing a series of substitution and/or deletion mutants to reduce the height of the T104-E108 loop. We have modelled the interactions between these SubB mutants and Neu5Gcα2–6 Galβ1–3Glc and predict that they would have improved recognition of α2–6-linked Neu5Gc without significantly impacting on α2–3-linked Neu5Gc binding, as shown in Fig. 2. We then constructed recombinant *subB* genes and expressed and purified the various proteins as C terminal His_6_-tagged fusion proteins from recombinant *E. coli* (see Materials and Methods). SubB proteins with single or double amino acid substitutions (T107A and S106A/T107A), a double deletion mutant (ΔS106/ΔT107) and a triple mutant (ΔS106/ΔT107/E108D) were successfully purified.

### Surface plasmon resonance of engineered SubB mutants

Purified SubB and the various mutant derivatives were then immobilized on Biacore chips and tested for binding affinities to a range of Neu5Ac- or Neu5Gc-terminating structures (free sialic acid, sialic acid-α2–3-lactose and sialic acid-α2–6-lactose), as well as to human and bovine α1-acid glycoprotein (AGP), by surface plasmon resonance (SPR) (Table 1). The human AGP glycans contain Neu5Ac^22, 23^ and the bovine AGP glycans contain both Neu5Ac and Neu5Gc^23^. The MS glycoproteomic analysis (Fig. S1) was performed to confirm the Neu5Ac and Neu5Gc distribution in the human and bovine AGP used in the SPR study. Wild-type SubB was found to have high affinity for α2–3-linked Neu5Gc-lactose and free Neu5Gc, as predicted from the glycan array result, with nanomolar binding affinities observed. No binding was observed for the α2–6-linked Neu5Gc-lactose (tested to a maximum concentration of 25 µM) and 2.2 µM affinity was observed for α2–3-linked Neu5Ac - a more than 300-fold decrease in binding compared to the equivalent Neu5Gc structure. The wild-type SubB also had a 13-fold reduced binding affinity for human AGP compared to bovine AGP. The wild-type SubB had no binding to any non-sialylated glycans tested (Table 1). The mutation in SubB_T107A_ had no significant effect on binding to any of the tested structures compared to the wild-type protein. SubB_S106A/T107A_ had improved binding to α2–6-linked structures, but this improvement was seen for both Neu5Ac and Neu5Gc. The nanomolar range affinities observed for all linked sugars tested including Neu5Acα2–8 (GT2; Table 1) and binding to sulfated Chondroitin (Chondroitin-6-sulfate; Table 1) reveals that SubB_S106A/T107A_ has a relaxed specificity range that included non-sialic acid structures. The SubB_ΔS106/ΔT107/E108D_ mutant had improved recognition of α2–6-linked Neu5Gc without changing the binding to the α2–6-linked Neu5Ac structures. However, the difference in affinity between α2–3-linked Neu5Ac and α2–3-linked Neu5Gc was reduced to 50-fold compared to the 300-fold observed for the wild-type. The SubB_ΔS106/ΔT107_ mutant was significantly improved for Neu5Gc vs Neu5Ac discrimination compared to the wild-type protein, and had the ability to bind α2–3-linked Neu5Gc and α2–6-linked Neu5Gc with binding affinities that were not significantly different between the two structures (15.3 nM vs 8.5 nM, respectively; *P* = 0.12). Thus, SubB_ΔS106/ΔT107_ exhibited the optimum combination of enhanced Neu5Gc vs Neu5Ac discrimination and the capacity to recognise both α2–3- and α2–6-linked Neu5Gc structures. SubB_ΔS106/ΔT107_ also demonstrated no binding to any of the non-sialylated glycans tested (Table 1). The anti-Neu5Gc antibody produced in chicken was used as a control and showed less selectivity and lower affinity for Neu5Gc containing glycans than any of the SubB proteins tested.

**Table 1:** Surface Plasmon Resonance analysis of Neu5Gc binding proteins.

SubB variant/antibody	Human α1-AGP	Bovine α1-AGP	Neu5Ac-α2–3-lac	Neu5Gc-α2–3-lac	Neu5Ac-α2–6-lac	Neu5Gc-α2–6-lac	Free Neu5Ac	Free Neu5Gc	Man5	maltose	Lactose	GT2	Chondroitin 6 sulfate
Anti-Neu5Gc antibody (IgY IgY)	*n.t*.	*n.t*.	249 ± 46 µM	2.34 ± 0.85 µM	*n.t*.	*n.t*.	NCDI	35.7 ± 4.2 µM	*n.t*.	*n.t*.	*n.t*.	*n.t*.	*n.t*.
Wild type SubB	2.12 ± 0.56 µM (Rmax = 125)	155.8 ± 22 nM (Rmax = 525)	2.24 ± 0.93 µM	6.62 ± 2.17 nM	NCDI	NCDI	NCDI	18.1 ± 5.9 nM	NCDI	NCDI	NCDI	NCDI	NCDI
S106A/T107A	723 ± 129 nM (Rmax = 142)	164 ± 10 nM (Rmax = 499)	489 ± 171 nM	1.52 ± 0.50 nM	348 ± 52 nM	8.05 ± 0.14 nM	3.27 ± 0.29 µM	6.61 ± 1.6 nM	NCDI	NCDI	NCDI	8.97 ± 2.2 µM	33.0 ± 7.6 µM
T107A	*n.t*.	*n.t*.	4.18 ± 1.6 µM	15.2 ± 0.02 nM	NCDI	208 ± 123 nM	NCDI	16.8 ± 0.99 nM	*n.t*.	*n.t*.	*n.t*.	*n.t*.	*n.t*.
ΔS106/ΔT107	1.65 ± 0.42 µM (Rmax = 7)	115 ± 37 nM (Rmax = 299)	NCDI	15.3 ± 5.8 nM	NCDI	8.53 ± 0.15 nM	NCDI	17.8 ± 4.0 nM	NCDI	NCDI	NCDI	NCDI	NCDI
ΔS106/ΔT107/E108D	2.82 ± 0.15 µM (Rmax = 165)	32.5 ± 2.6 nM (Rmax = 276)	371 ± 64 nM	7.39 ± 0.72 nM	NCDI	3.45 ± 0.87 nM	NCDI	45.1 ± 1.2 nM	*n.t*.	*n.t*.	*n.t*.	*n.t*.	*n.t*.

Binding affinities of wild type SubB, various mutant derivatives and an anti-Neu5Gc IgY antibody, to purified tri- and monosaccharides and/or human or bovine α1-acid glycoprotein (AGP) was determined by SPR, as described in the Materials and Methods. NCDI indicates that no concentration- dependent interaction was observed with concentrations ranging up to 100 µM; *n.t*.: Not tested; Rmax: the total amount of response units (RUs) of the analyte bound to the protein (the higher the number the more the glycan/glycoprotein was bound by the immobilised SubB).

### Glycan array analysis of wild-type SubB, SubB_S106A/T107A_ and SubB_ΔS106/ΔT107_

To assess whether the preferred, Neu5Gc-specific SubB_ΔS106/ΔT107_ mutation introduced specificity for non-sialylated structures, not covered by the SPR analysis, glycan array analysis was performed on the SubB wild-type, SubB_ΔS106/ΔT107_ and SubB_S106A/T107A_ mutants (Table S1). Wild-type SubB displayed significant binding to only four of 402 structures on the glycan array; Neu5Gcα2–3 Gal, Neu5Gcα2–3 Galβ1–4GlcNAc and two Neu5Acα2–3Galβ1–4GlcNAc terminated structures. This is in agreement with previously published glycan array analysis of SubB^8^ (www.functionalglycomics.org/glycomics/HServlet?operation=view&sideMenu=no&psId=primscreen_1579#). SubB_ΔS106/ΔT107_ only had displayed significant binding to four structures on the array. These were limited to structures terminating with Neu5Gcα2–3Gal or Neu5Gcα2–6 Gal. SubB_S106A/T107A_ bound to 18 glycans in total on the array including structures containing Neu5Gc and Neu5Ac. It also recognised sulfated structures including glycosaminoglycans (heparin and chondroitin-6-sulfate) and sulfated lactosamine structures (Table S1). SubB_S106A/T107A_ also recognised a range negatively charged of monosaccharides (Neu5Ac, Neu5Gc, 9-NAc-Neu5Ac, 3-O-Su-GlcNAc) on the array.

### ELISA of engineered SubB against human and bovine proteins/serum

**Figure 3:**
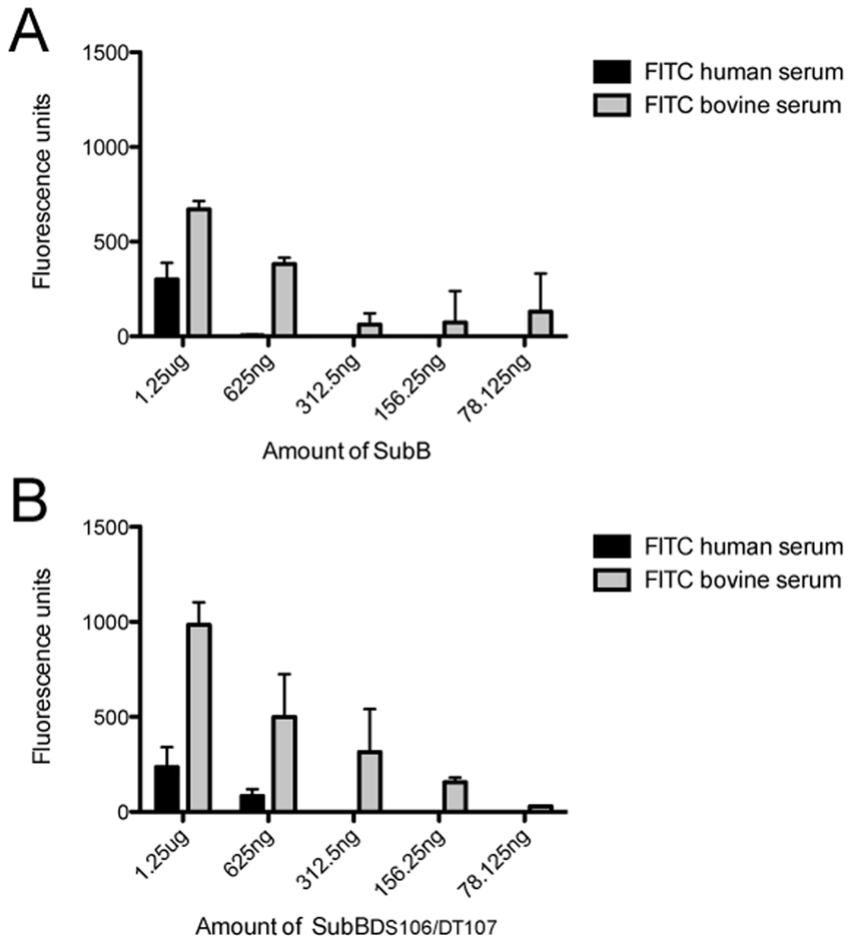
ELISA of engineered SubB against FITC-labelled human and bovine serum. SubB (**A**) and SubB_ΔS106/ΔT107_ (**B**) coated onto ELISA plates was able to capture FITC-labelled human and bovine serum proteins. Error bars show +1 SD from the mean of duplicate assays.

To assess the ability of the engineered mutants to detect the presence of Neu5Gc in biological samples ELISA assays were performed. Using dishes coated with a dilution series of SubB, labelled serum proteins from human and bovine sources were tested. A two-fold improvement in differential recognition of the Neu5Gc containing serum proteins from bovine was identified with SubB_ΔS106/ΔT107_ (Fig. 3).

### Detection of human vs bovine AGP

**Figure 4:**
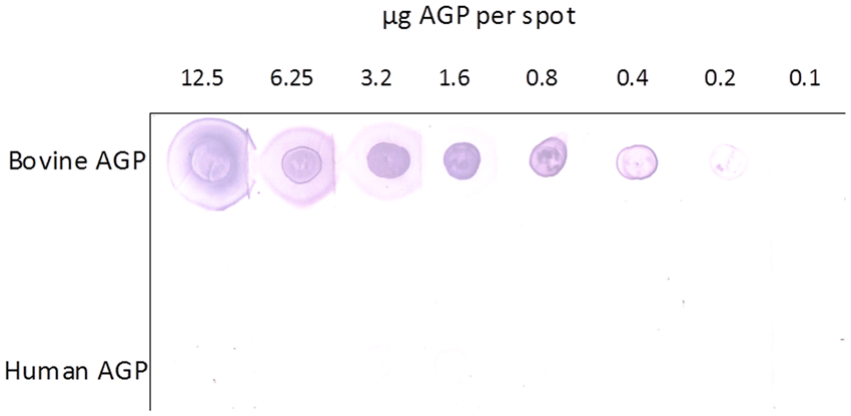
Lectin overlay assay. Binding of SubB_ΔS106/ΔT107_ to serial dilutions of human or bovine AGP spotted onto nitrocellulose (total amounts of protein per spot indicated), was determined as described in the Materials and Methods.

To independently verify the capacity to discriminate between human and bovine AGP (only bovine AGP displays significant levels of Neu5Gc-terminating glycans), serially diluted glycoproteins were spotted onto nitrocellulose filters and after washing and blocking, filters were overlayed with purified biotinylated SubB_ΔS106/ΔT107_. Bound lectin was then detected on washed filters using Streptavidin-AP (Fig. 4). SubB_ΔS106/ΔT107_ binding to bovine AGP was detectable down to approximately 200 ng/spot, while significant binding to human AGP was not detectable even at the maximum amount tested (12.5 μg/spot). This discriminatory power is consistent with the SPR data above.

## Discussion

Neu5Gc is an important diagnostic and prognostic marker in human carcinomas, with elevated Neu5Gc expression detected in breast, ovarian, prostate, colon and lung cancer^11, 12^. Wild-type SubB had unprecedented specificity for glycans terminating in Neu5Gc, but bound poorly to α2–6-linked Neu5Gc and still recognised α2–3-linked Neu5Ac structures albeit weakly^8^. To improve the recognition of SubB for α2–6-linked Neu5Gc and make it more specific for Neu5Gc, we engineered SubB using structure-aided modifications, with specific focus on the T104-E108 loop.

Manipulation of this loop had two specific outcomes through the modification of the same two amino acids. Firstly, alanine substitution of S106 and T107 (S106A/T107A) led to a loss of specificity for Neu5Gc, producing a lectin capable of binding to all tested terminally sialylated glycans regardless of linkage (α2–3, α2–6 and α2–8) or sialic acid type (Neu5Ac or Neu5Gc) in SPR studies. Glycan array analysis confirms the relaxed specificity and revealed binding to additional, sulfated glycans. The second was that deletion of the same two amino acids (ΔS106/ΔT107) produced a lectin with exquisite specificity for Neu5Gc regardless of linkage (α2–3 and α2–6). The SubB_ΔS106/ΔT107_ mutant was significantly improved for the recognition Neu5Gc containing structures compared to the wild-type SubB. SubB_ΔS106/ΔT107_ also had no difference in its ability to bind α2–3-linked Neu5Gc or α2–6 linked Neu5Gc structures, making it a significant improvement over the wild-type protein. Further modifications of the SubB protein outside of the S106 and T107 amino acids produced no significant improvement in specificity. The SubB_ΔS106/ΔT107/E108D_ mutant protein, which is the SubB_ΔS106/ΔT107_ protein with a E108D mutation also added, was less able to distinguish α2–3-linked Neu5Gc from α2–3-linked Neu5Ac than SubB_ΔS106/ΔT107_ and had stronger binding to the human α1-Acid glycoprotein than the SubB_ΔS106/ΔT107_ mutant (24 fold more protein bound by SubB_ΔS106/ΔT107/E108D_ than SubB_ΔS106/ΔT107_).

These improved SubB mutants offer a new tool for the testing of biological samples, particularly serum and other fluids from individuals with cancer or suspected of having cancer.

## Methods

### Structural modeling of SubB

The three-dimensional structure of the SubB mutants were modeled using Phyre2^24^. Neu5GCα2–6Galβ1–3Glc was acquired from PDB ID: 4EN8^25^ and modeled into the SubB and SubB mutant structures manually using Coot^26^.

### Construction and expression of SubB mutants

Mutations were introduced into the *subB* coding sequence (close to the 3′ end) by direct high-fidelity PCR using the forward primer pETSubBF and the respective mutant-specific reverse primers listed in Table 2. PCR products were cloned into the *Bam*HI and *Xho*I sites of pET-23(+) (Novagen) and transformed into *E. coli* BL21(DE3). SubB derivatives were expressed and purified as His_6_-tagged fusion proteins by Ni-NTA affinity chromatography, as previously described^4^. Proteins were >95% pure as judged by SDS-PAGE and Coomassie blue staining.

**Table 2:** Oligonucleotides.

Primer	Sequence 5′–3′
pETSubBF	TTGTAAGGATCCGGAGGTGCATATGACG
pETSubB_T107A_R	GATTATCTCGAGTGAGTTCTTTTTCCTGTCAGGACCAAAACATTCTGCCGATGTGGTGCAGGTTG
pETSubB_S106A/T107A_R	GATTATCTCGAGTGAGTTCTTTTTCCTGTCAGGACCAAAACATTCTGCCGCTGTGGTGCAGGTTG
pETSubB_ΔS106/ΔT107_R	GATTATCTCGAGTGAGTTCTTTTTCCTGTCAGGACCAAAACATTCTGTGGTGCAGGTTGATAACCC
pETSubB_ΔS106/ΔT107/E108D_R	GATTATCTCGAGTGAGTTCTTTTTCCTGTCAGGACCAAAACAGTCTGTGGTGCAGGTTGATAACCC

### Surface Plasmon Resonance of SubB and engineered SubB mutants

Surface Plasmon resonance (SPR) was run using the Biacore T100 system (GE) as described previously^27^. Briefly, SubB, SubB mutants and anti-Neu5Gc IgY (SiaMab; formerly Sialix/GC-Free Inc., San Diego, CA, USA) were immobilized onto flow cell 2–4 of a series S sensor chip CM5 (GE) using the NHS capture kit and flow cell 1 was run as a blank immobilization. Monosaccharides, disaccharides, oligosaccharides and α1-Acid glycoprotein from human and bovine sources (Sigma-Aldrich; See Table 1) were flowed over at 0.01–100 µM on initial range finding experiments. Concentrations were adjusted and all data were analysed using single cycle kinetics using the Biacore T100 Evaluation software.

### Mass spectrometric analysis of α1-Acid glycoprotein

AGP from human plasma (Sigma-Aldrich G9885) and bovine plasma (Sigma-Aldrich G3643) (1 mg in 6 M guanidinium chloride, 50 mM Tris-HCl pH 8) was reduced and alkylated with 10 mM dithiothreitol and 25 mM acrylamide, respectively. Protein was then precipitated by adding 4 volumes of 1:1 methanol:acetone, incubating in −20 °C for 16 h and then centrifuged (18,000 rcf, 10 min) to collect the pellet. The precipitated protein was resuspended in 50 µL of 50 mM Tris-HCl pH8 and digested (37 °C, 16 h) with 1 µg trypsin (Trypsin Gold, Promega). Digested peptides were then desalted with C18 ZipTips (Millipore).﻿ Mass spectrometry of desalted peptides was performed using a TripleT of 5600 instrument (SCIEX) as previously described [﻿﻿Zacchi Schulz MCP 2016 PMID: 27094473 doi:10.1074/mcp.M115.056366﻿].

### Glycan array analysis of SubB and engineered SubB mutants

Glycan array slides were printed on SuperEpoxy 3 (Arrayit) activated substrates using an Arrayit Spotbot Extreme contact printer as previously described^28^. For each subarray 2 μg of SubB proteins were pre-complexed with anti-His tag antibody (Cell signalling) and Alexa555 secondary and tertiary antibodies (rabbit anti-mouse; goat anti-rabbit) at a ratio of 2:1:0.5:0.25 in a final volume of 500 μL. This 500 μL antibody protein complex was added to a 65 μL gene frame (Thermo Scientific) without a coverslip. Washing and analysis was performed as previously described^27^.

### ELISA analysis of SubB and the engineered SubB_ΔS106/ΔT107_ mutant

Wells of black 96-well NUNC Maxisorp plates were coated with SubB or SubB_ΔS106/ΔT107_ protein two-fold serially diluted in 100 mM bicarbonate/carbonate coating buffer (pH 9.6) starting at 1.25 μg of protein overnight at 4 °C. Wells were washed 3 times with phosphate-buffered saline, 0.05% Tween-20 (PBS-T) before blocking solution (3% BSA) was added for 1 hour at room temperature. Proteins in normal human serum and bovine serum were fluorescently labelled by combining neat serum with 100 µM FITC dye (Peirce) and incubating on ice for 1 hour. Excess dye was removed using a 1 kDa size exclusion spin column. 100 μl of FITC-labelled normal human serum or bovine serum was added to wells coated with SubB or SubB_ΔS106/ΔT107_ and wells were incubated for 1 hour at room temperature. Wells were washed 3 times with PBS-T. 100 μl of PBS was added to each well before the fluorescence was measured at 485/535 nm. Fluorescence unit values are shown as the mean of duplicates +/−SD, with the mean fluorescence units obtained for wells containing all reagents except for the SubB proteins subtracted. Any negative value was considered as 0.

### SubB overlay experiments

Purified SubB_ΔS106/ΔT107_ was labelled with biotin using the EZ-Link^®^ Sulfo-NHS-Biotinylation Kit (Thermo Scientific) according to the manufacturer’s instructions. Purified human and bovine α−1 acid glycoprotein (Sigma cat. nos G9885 and G3643) were dissolved in water at 5 mg/ml and 5 μl volumes of serial two-fold dilutions were spotted onto nitrocellulose filters and air dried at 37 °C overnight. Filters were then blocked with 5% skim milk in Tris-buffered saline with 0.05% Tween 20 (TTBS) for 2 h. After washing three times in TTBS, filters were overlaid with 1 μg/ml biotin-SubB_ΔS106/ΔT107_ in TTBS and incubated overnight at 4 °C. Filters were then washed three times in TTBS and bound biotin-SubB_ΔS106/ΔT107_ was detected using streptavidin-alkaline phosphatase conjugate (Roche). Filters were developed using a chromogenic nitro-blue tetrazolium/X-phosphate substrate system (Roche).

## Electronic supplementary material

Figure S1

Table S1
