# Supplementary material for: Structure aided design of a Neu5Gc specific lectin
Source: Sci Rep. 2017 May 4;7:1495. doi: 10.1038/s41598-017-01522-9 (PMC5431456; doi:10.1038/s41598-017-01522-9)
Supplement: Supplementary file 1 — Figure S1 [file 41598_2017_1522_MOESM1_ESM.doc]

**Structure aided design of a Neu5Gc specific lectin.**

Christopher J. Day^a#^, Adrienne W. Paton^b#^, Melanie A. Higgins^b^, Lucy K. Shewell^a^, Freda E.-C. Jen^a^, Benjamin L. Schulz^c^, Brock P. Herdman^b^, James C. Paton^b*^, Michael P. Jennings^a*^.

**Supplementary**


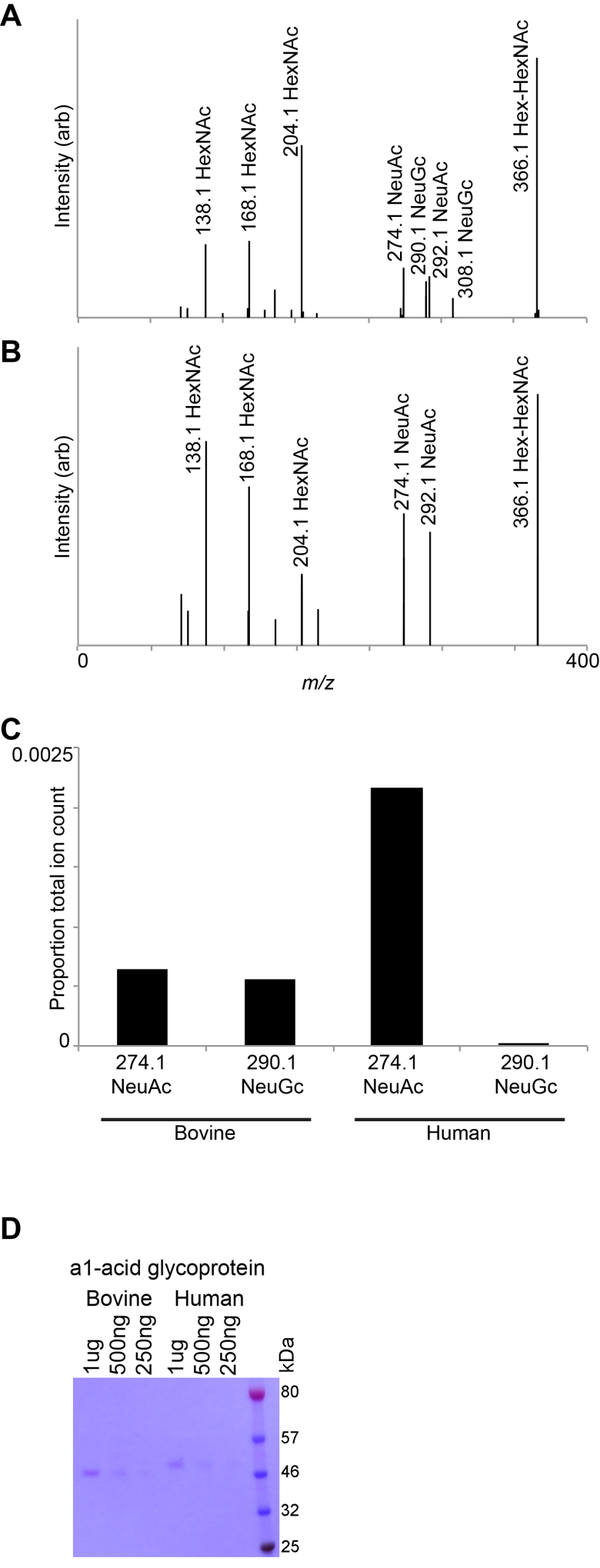


**Figure S1. NeuAc and NeuGc oxonium ions from human and bovine alpha-1-acid glycoprotein tryptic digests.** Low mass region of MS/MS spectra of (A) glycopeptide ion at an *m/z* of 1183.16^3+^ corresponding to peptide TFMLAASWN[Hex_2_HexNAc_2_NeuAc_1_NeuGc_1_+Man_3_GlcNAc_2_]GTK from bovine alpha-1-acid glycoprotein and (B) glycopeptide ion at an *m/z* of 1122.28^4+^ corresponding to peptide QDQCIYN[Hex_3_HexNAc_3_NeuAc_2_+Man_3_GlcNAc_2_]TTYLNVQR from human alpha-1-acid glycoprotein showing abundant oxonium ions, including NeuAc-specific 274.1 and 292.1, and NeuGc-specific 290.1 and 308.1. (C) Intensity of NeuAc- and NeuGc-specific oxonium ions as a proportion of the total ion intensity from all MS/MS spectra from LC-MS/MS analysis of human and bovine alpha-1-acid glycoprotein tryptic digests. (D) Protein gel of the human and bovine AGP used in MS, ELISA, Biacore and dot blot.
